# Dr. John Hudson Grant

**Published:** 1914-09

**Authors:** 


Dr· John Hudson Grant, Georgetown, 1890, died at his residence in New York City, July 26. Dr. Grant was formerly a resident of Buffalo and had many friends in western New York. He was a Civil War veteran, retired as a hospital steward. After graduating in medicine, he was appointed Deputy Commissioner of Agriculture for the State of N. Y. and, in connection with his official duties, spent some years in Buffalo, being then transferred to Albany and finally to New York City.

